# Exploring the Male Perspectives Regarding the Need for Postpartum Contraception in Their Female Partners: A Cross-Sectional Study Among Husbands in India

**DOI:** 10.7759/cureus.108882

**Published:** 2026-05-15

**Authors:** Neelima Choudhary, Avir Sarkar, Sneha Kumari, Malvika Dixit, Vartika Sharma, Suman S Samal, Souvik Das

**Affiliations:** 1 Obstetrics and Gynaecology, Employees State Insurance Corporation (ESIC) Medical College and Hospital, Faridabad, IND; 2 Obstetrics and Gynaecology, Noida International Institute of Medical Sciences, Noida, IND; 3 Obstetrics and Gynaecology, All India Institute of Medical Sciences, Jodhpur, Jodhpur, IND; 4 Obstetrics and Gynaecology, All India Institute of Medical Sciences, Kalyani, Kalyani, IND

**Keywords:** contraception, contraceptive choice, methods of contraception, postpartum contraception, unmet need for contraception

## Abstract

Background

Postpartum contraception is vital for maternal and neonatal health. In India, male partners play a decisive role in reproductive choices, yet their perspectives on postpartum contraception remain underexplored. Understanding men’s knowledge, beliefs, fears, attitudes, and practices is crucial for improving contraceptive uptake and promoting shared decision-making in family planning. We aimed to design a study to explore Indian men’s perspectives regarding postpartum contraception.

Materials and methods

A cross-sectional, questionnaire-based study was conducted over one year at a tertiary care teaching hospital in India after obtaining institutional ethics approval. A total of 1560 husbands of antenatal and postpartum women were recruited after informed consent. A semi-quantitative 20-item questionnaire assessed five domains: knowledge, beliefs, fears, attitudes, and practices. Data were analyzed using SPSS version 25, with descriptive statistics and chi-square tests; *p*<0.05 was considered significant.

Results

The mean participant age was 31.2±4.9 years, with near-equal urban and rural representation. While 76.1% were aware of the need for birth spacing, only 48.6% recognized early return of fertility postpartum. About 83.6% believed contraception was necessary, and 70.5% considered it safe for their wives. However, misconceptions persisted: 39.7% feared adverse effects on lactation and 35.4% on infant growth. Although 73.2% desired involvement in contraceptive decision-making, practices remained female-centric; intrauterine contraceptive device (IUCD: 28.3%) and tubal ligation (23.9%) were preferred for partners, while only 14.4% of men expressed willingness for vasectomy.

Conclusion

Indian men increasingly acknowledge the importance of postpartum contraception but continue to harbor misconceptions about safety and fertility. Their supportive attitudes contrast with the limited adoption of male methods, reflecting persistent gendered norms. Integrating husbands into antenatal counselling, correcting misconceptions, and promoting male responsibility are key to enhancing contraceptive uptake and achieving equitable family planning outcomes.

## Introduction

Postpartum contraception is central to reproductive health and family welfare. The World Health Organization (WHO) recommends an inter-pregnancy interval of at least 24 months to reduce maternal and neonatal morbidity and mortality [1]. Closely spaced pregnancies are associated with higher risks of anemia, preterm birth, low birth weight and maternal complications [1]. In India, with its high fertility and significant unmet need for contraception, the postpartum period provides an important opportunity to initiate family planning [2].

Male partner perspectives, however, strongly influence contraceptive uptake. In many Indian households, particularly those of lower socioeconomic status, men are primary decision-makers regarding reproductive choices. Male hesitancy, rooted in cultural norms, inadequate knowledge, and misconceptions about contraceptive safety, has been identified as a barrier to the adoption of postpartum contraception [2]. At the same time, increasing evidence shows that the inclusion of men in family planning discussions improves contraceptive continuation, strengthens reproductive autonomy, and reduces unintended pregnancies [3,4].

Despite global recognition of the importance of male involvement, limited Indian research has systematically examined men’s perceptions of postpartum contraception. The existing literature often focuses on women’s knowledge and practices, leaving a gap in understanding the male perspective. This omission is critical as effective family planning requires not just female acceptance but also male support.

Recent reviews emphasize the need for couple-based interventions, beginning during antenatal care, to ensure sustained uptake of contraception after delivery [2,3]. Male involvement in counselling can help dispel myths, address concerns regarding lactation, infant health, or sexual satisfaction, and foster joint decision-making. Given these considerations, we designed this study to evaluate the knowledge, beliefs, fears, attitudes, and practices of Indian husbands regarding postpartum contraception.

## Materials and methods

This was a cross-sectional, descriptive, questionnaire-based study conducted in a tertiary care teaching institute and hospital in northern India, over a period of one year, following institutional ethics committee clearance. It assessed the perception of husbands regarding postpartum contraception for pregnancy spacing after delivery by their wives, specifically exploring their knowledge, beliefs, fears, attitudes, and practices.

The study participants included husbands of women attending third-trimester antenatal visits in the outpatient departments or admitted to the labor wards for delivery. Every consecutive parturient woman's husband was recruited until there was a negative consent to participate. Recruitment was also done during routine visits in the postpartum clinics for suture removal after cesarean delivery or for neonatal check-up. All men recruited were older than 18 years of age and had given written informed consent for participating in this study. We excluded participants younger than 18 years of age, individuals with severe cognitive impairment or psychiatric illness precluding consent, couples with male factor infertility (conception through donor sperm), and couples in whom further pregnancy was medically contraindicated (e.g., WHO category 4 heart disease, because there would be a selection bias as they were compelled to accept contraception for medical safety) [1].

Based on a prior study by Williams et al. and taking 95% confidence interval (CI), 80% power, and 20% attrition, the minimum required sample size was 468 [3]. Considering the huge patient load and to enhance the validity of our results, we recruited 1560 participants.

A semi-quantitative, self-administered questionnaire was designed with 20 items across five domains: knowledge, beliefs, fears, attitudes, and practices (as these were the areas to be explored in this study). The questionnaire was designed in English and Hindi. The tool was initially piloted among 50 participants. Cronbach’s alpha was 0.78, indicating acceptable reliability. Thereafter, the questionnaire was used to collect data from all the recruited participants.

Participants were approached in outpatient clinics, labor wards, and postpartum follow-up clinics. After informed consent, questionnaires were completed privately, with assistance provided by the trained staff of the department, if required. The primary outcome was the prevalence of knowledge and beliefs about postpartum contraception. The secondary outcomes included perceived fears, attitudes toward contraceptive methods, and the intended practices.

Data was entered into Microsoft Excel (Microsoft, Redmond, WA) and analyzed using SPSS version 25 (IBM Corp., Armonk, NY). Descriptive statistics were reported as mean±standard deviation (SD) for continuous variables and proportions for categorical variables.

## Results

Of the total 1560 participants, the mean age was 31.2±4.9 years. Most of the participants (62.4%) were aged between 25 and 34 years. Urban and rural representation was nearly equal. Almost half of the recruited population had a graduation degree, while 25.3% were postgraduates. Skilled/semi-skilled occupations were more common than unskilled/informal work, as shown in Table 1.

**Table 1:** Demographic characteristics of participants (n=1560)

Characteristic	n (%)
Age (years)	
<25	184 (11.8)
25-34	973 (62.4)
35-44	343 (22.0)
≥45	60 (3.8)
Residence	
Urban	760 (48.7)
Rural	800 (51.3)
Education	
Secondary or less	446 (28.6)
Graduate	719 (46.1)
Postgraduate	395 (25.3)
Occupation	
Skilled/semi-skilled	897 (57.5)
Unskilled/informal	663 (42.5)

Knowledge

Analysis of participants’ knowledge (Table 2) revealed that while three-quarters of respondents (76.1%) were aware of the concept of pregnancy spacing, fewer than half (48.6%) recognized the possibility of early return of fertility postpartum. A little over half (55.2%) could identify at least one method of postpartum contraception, indicating moderate awareness. However, a significant proportion (58.1%) considered lactational amenorrhea an unreliable method, suggesting a gap in accurate understanding. Overall, these findings indicate that although the importance of spacing is acknowledged, specific knowledge about the timing of fertility return and contraceptive options remains incomplete.

**Table 2:** Knowledge about postpartum contraception

Question	Yes (n (%))	No (n (%))
1. Aware of birth spacing	1187 (76.1)	373 (23.9)
2. Believe spacing important	1269 (81.3)	291 (18.7)
3. Know earliest return of fertility	758 (48.6)	802 (51.4)
4. Know ≥1 postpartum contraceptive method	861 (55.2)	699 (44.8)
5. Consider lactational amenorrhea reliable	654 (41.9)	906 (58.1)

Beliefs

Belief patterns (Table 3) showed encouraging trends, with 83.6% agreeing that contraception is needed after childbirth. Regarding safety, 72.9% believed contraception methods were safe for themselves and 70.5% considered them safe for their wives. However, concerns persisted regarding infant safety, with only 65.8% of the participants being confident that contraception would not harm the newborn. This highlights that while men largely accept the necessity and maternal safety of contraception, reservations about neonatal safety remain an important barrier.

**Table 3:** Beliefs regarding postpartum contraception

Question	Yes (n (%))	No (n (%))
6. Believe contraception needed postpartum	1304 (83.6)	256 (16.4)
7a. Safe for self	1138 (72.9)	422 (27.1)
7b. Safe for wife	1100 (70.5)	460 (29.5)
7c. Safe for newborn	1026 (65.8)	534 (34.2)

Fears

Perceived fears (Table 4) demonstrated substantial reservations among men. About 42.1% worried that contraception required frequent hospital visits, while 39.7% feared potential effects on lactation. Concerns regarding the baby’s growth (35.4%) and partner’s health (37.8%) were also common. Notably, nearly 30% believed contraception might impair future fertility, and 28.9% expressed apprehension about reduced sexual pleasure. These findings underline the role of misconceptions and misinformation in shaping negative attitudes toward postpartum contraceptive adoption.

**Table 4:** Fears regarding postpartum contraception

Concern	Yes n (%)	No n (%)
8. Frequent hospital visits required	657 (42.1)	903 (57.9)
9a. May affect lactation	619 (39.7)	941 (60.3)
9b. May affect baby’s growth	552 (35.4)	1008 (64.6)
9c. May affect future fertility	462 (29.6)	1098 (70.4)
9d. May affect partner’s health	590 (37.8)	970 (62.2)
9e. May reduce sexual pleasure	451 (28.9)	1109 (71.1)

Attitudes

Attitudinal assessment (Table 5) reflected a generally supportive outlook. While most participants preferred female-dependent methods (61.2%), nearly 80% expressed willingness to support their partner’s use of contraception. Timing of counselling emerged as a critical factor, with 42.6% preferring discussions during pregnancy. Effectiveness (61.3%) was cited as the most important factor influencing choice, followed by safety and ease of use. Encouragingly, nearly three-quarters (73.2%) wanted to be involved in decision-making, and two-thirds (67.9%) favored initiation before hospital discharge. These findings suggest that men are open to involvement when engaged at the right time.

**Table 5:** Attitudes toward postpartum contraception LAM: Lactational amenorrhea method; LARC: Long-acting reversible contraception.

Question	Response	n (%)
10. Preference for method type	Male-dependent	605 (38.8)
	Female-dependent	955 (61.2)
11. Support partner’s use (beyond LAM)	Yes: 1238 (79.4)	No: 322 (20.6)
12. Preferred time for discussion	During pregnancy: 665 (42.6)	At admission: 342 (21.9)
13. Factors influencing choice	Efficacy: 957 (61.3)	Safety: 365 (23.4)
14. Desire inclusion in decision-making	Yes: 1141 (73.2)	No: 419 (26.8)
15. Desire initiation before discharge	Yes: 1060 (67.9)	No: 500 (32.1)
16. Preferred type of method	LARC: 872 (55.9)	Short-acting: 688 (44.1)

Practices

An analysis of contraceptive practices (Table 6) demonstrated that over half of the respondents (56.4%) preferred consulting an obstetrician or gynaecologist before deciding on postpartum contraception, while just over a quarter (26.4%) relied on their partner’s input. Very few (5.5%) reported friends as a source of advice, and none relied on midwives, underlining the medicalized nature of contraceptive counselling in this setting. In terms of personal contraceptive practices, barrier methods such as condoms were the most popular (37.5%), followed by vasectomy after family completion (14.6%), and reversible inhibition of sperm under guidance (RISUG; 8.2%). Notably, 30% reported unwillingness to use any method. When asked about preferred methods for their partners, intrauterine contraceptive device (IUCD: 28.3%) and tubal ligation (23.9%) dominated, followed by pills (17.6%). Interestingly, nearly one-fifth (19.6%) expressed willingness to leave the decision to their wives. Despite this, male sterilization acceptance was extremely low, with only 14.4% indicating willingness to undergo vasectomy in the future, reflecting persistent gendered contraceptive responsibility norms.

**Table 6:** Practices of participants regarding postpartum contraception (n=1560)

Question	Response Options	n (%)
17. Who would you like to consult prior to choosing postpartum contraception?	Partner	412 (26.4)
	Friends	86 (5.5)
	Family Members	182 (11.7)
	Midwife	0 (0.0)
	Obstetrician & Gynaecologist	880 (56.4)
18. Which method would you choose for yourself?	None	468 (30.0)
	Withdrawal method	152 (9.7)
	Barrier (Condoms)	585 (37.5)
	RISUG (Reversible inhibition of sperm under guidance)	128 (8.2)
	Vasectomy (after family completion)	227 (14.6)
19. Which method would you choose for your partner?	None	148 (9.5)
	Calendar method	168 (10.8)
	Pills	274 (17.6)
	Injections	176 (11.3)
	Intrauterine contraceptive device (IUCD)	442 (28.3)
	Tubal ligation (after completion of family)	373 (23.9)
	Whatever she chooses	306 (19.6)
20. Do you want to undergo vasectomy after the completion of family in future?	Yes	225 (14.4)
	No	1335 (85.6)

## Discussion

The present study explored male perspectives on postpartum contraception, a subject that has historically been under-researched in developing countries, including India [1,2]. With a large sample size of 1560 participants, our findings provide meaningful insights into the knowledge, beliefs, fears, attitudes, and practices of husbands regarding contraceptive use by their female partners in the postpartum period.

Our results highlight that while general awareness of the importance of birth spacing was high, only about half of the respondents could correctly identify contraceptive methods suitable for postpartum use. This indicates that although men conceptually endorse spacing, their knowledge is often superficial and fragmented. The persistence of misconceptions about lactational amenorrhea being ineffective reflects the ongoing need for targeted education. Beliefs were largely positive, with over 80% agreeing on the necessity of postpartum contraception, yet fears about lactation, neonatal safety, and hospital visits suggest that anxiety and misinformation still influence decision-making.

Attitudes were promising, with a majority willing to support their partners in adopting contraception, and nearly three-quarters wanting to be included in decision-making. However, practices demonstrated a reliance on female-dependent methods, especially IUCD and sterilization, with limited willingness to adopt male-centered options such as vasectomy (14.4%). This reflects entrenched gender norms that place the burden of contraception on women.

Our findings resonate with earlier Indian studies showing that male awareness of contraception has increased over time but remains inconsistent regarding specific methods [5]. A 2022 study in Maharashtra also noted that although men supported spacing, misconceptions about side effects reduced actual uptake [6]. Similar trends were observed in sub-Saharan Africa, where husbands’ limited knowledge and fears regarding adverse effects constrained contraceptive adoption despite overall approval of family planning [7].

In Saudi Arabia, Sait et al. reported that only 43% of men were aware of modern contraceptive methods, much lower than the awareness in our cohort, suggesting that contextual cultural differences influence knowledge [4]. In contrast, studies from Bangladesh and Nepal have highlighted stronger male resistance to female contraceptive use, underscoring that Indian men may be relatively more supportive when adequately counselled [8].

Globally, misconceptions regarding postpartum contraception persist. A systematic review on male involvement in postpartum family planning showed that fears of impaired lactation, compromised fertility, and reduced sexual satisfaction were consistently reported across diverse settings [9]. Our study confirms these fears among Indian men, suggesting they are universal barriers that must be addressed through couple-centered counselling.

Male attitudes in our cohort were encouraging, with 73.2% desiring involvement in decision-making. This aligns with findings from Nigeria, where male engagement during antenatal visits significantly improved postpartum contraceptive uptake [10]. Yet, the reluctance toward vasectomy mirrors national data showing vasectomy rates declining from 3.5% in the 1990s to less than 0.5% in the National Family Health Survey-5 (NFHS-5) (2019-21) [11-16]. The persistence of female sterilization as the dominant permanent method in our study further underscores this imbalance.

Strengths and limitations

The strength of this study lies in its large sample size, robust questionnaire covering multiple dimensions, and inclusion of men across antenatal, intrapartum, and postpartum periods. This ensured diversity of perspectives across different stages of pregnancy and parenthood. The pilot testing and use of Cronbach’s alpha for validation added methodological rigor.

However, limitations must be acknowledged. First, being hospital-based, our sample may not represent men from rural or underserved populations who do not access institutional maternity care. Second, self-reported data are prone to social desirability bias; men may have over-reported supportive attitudes toward contraception. Third, the cross-sectional design precludes establishing causal associations between knowledge, beliefs, and practices. Finally, the reliance on hypothetical responses (e.g., willingness to use vasectomy) may not translate into real-world adoption.

Policy and practice implications

Despite these limitations, our findings hold significant implications. First, antenatal clinics should routinely involve husbands in counselling sessions, as our data suggest that men are most receptive to discussions during pregnancy. Second, interventions must focus on correcting misconceptions, particularly regarding lactation and neonatal safety. Third, programs should promote shared decision-making rather than placing the onus solely on women. Fourth, national family planning campaigns must reframe male contraception, especially vasectomy, to challenge the prevailing gender norms. Evidence from Rwanda and Ethiopia has shown that when men are directly engaged, contraceptive prevalence rises significantly [2,7]. Similar strategies could be adapted in the Indian context.

Future studies should employ longitudinal designs to examine whether supportive male attitudes translate into sustained contraceptive use by couples. Qualitative research is also needed to understand deeper cultural and psychosocial barriers to male contraceptive adoption.

## Conclusions

Indian men demonstrate a growing recognition of the need for postpartum contraception, but misconceptions and fears persist, particularly around lactation and infant safety. While attitudes toward shared decision-making is positive, reliance on female-dependent methods remains dominant and male sterilization remains highly resisted.

Strengthening male involvement through targeted counselling during antenatal visits, addressing misconceptions and promoting male responsibility in contraception are critical steps to enhance uptake of postpartum contraception and support family welfare in low- and middle-income countries like India.
